# Obesity inducing acute respiratory distress syndrome: we should choose the right population!

**DOI:** 10.1186/s13054-019-2631-2

**Published:** 2019-10-28

**Authors:** Patrick M. Honore, Leonel Barreto Gutierrez, Sebastien Redant, Keitiane Kaefer, Andrea Gallerani, David De Bels

**Affiliations:** ICU Department, Centre Hospitalier Universitaire Brugmann- Brugmann University Hospital, Place Arthur Van Gehuchtenplein, 4, 1020 Brussels, Belgium

We read with interest the article of Palakshappa et al. which demonstrate that low levels of adiponectin were not associated with an increased risk of acute respiratory distress syndrome (ARDS) in patients with severe sepsis and septic shock [1] going against the hypothesis that low levels of adiponectin are the mechanism explaining the association of obesity with ARDS. It is unclear whether circulating adiponectin is involved in the pathogenesis of ARDS or simply represents an epiphenomenon. According to Rubenfeld et al., the most common risk factor from either a pulmonary or non-pulmonary source that accounts for 79% of ARDS cases is sepsis [2] and sepsis represents 80% of the risk factors for ARDS, and knowing that sepsis was present in 100% of the patients in this study [1], it is highly probable that sepsis is blunting the potential effects of obesity as a mechanism triggering ARDS in obese patients. Other risk factors include aspiration, lung contusion/trauma, acute pancreatitis, blood product transfusion, burn injury/smoke inhalation, cardiopulmonary bypass, and indeed obesity [2]. In addition, if we look carefully to the baseline characteristics of both populations with and without ARDS, some differences are intriguing. Regarding the incidence of chronic liver disease, we have 4% in the non-ARDS group vs 19% in the ARDS group (*P* < 0.009). It stands to reason that sicker patients have more risks of developing multiple organ failure including ARDS [3]. Also when looking at the Acute Physiology and Chronic Health Evaluation (APACHE) III score, with a more severe APACHE III being at risk of ARDS, it was 90 for non-ARDS vs 98 for ARDS (*P* < 0.003) [3]. Regarding the pulmonary source of sepsis, it was respectively 28% for non-ARDS versus 52% for ARDS (*P* < 0.02). Once again, a pulmonary source of sepsis can be more easily treated by antibiotics, whereas an extra-pulmonary source of sepsis is more difficult to find and to control. Uncontrolled sepsis is a major risk factor for ARDS [4]. Adiponectin, an anti-inflammatory adipokine, is reduced in sepsis [5]. This low level of adipokine in sepsis may be a bias in this study as sepsis may further lower adipokine [5]. Altogether, confirming or not this relationship by choosing obese patients without sepsis would be an interesting study as sepsis could blunt the risk factor of obesity for ARDS. Other confounders should be eliminated for a further study as well.

## Data Availability

Not applicable.

## Abbreviations

ARDS: Acute respiratory distress syndrome
APACHE III: Acute Physiology and Chronic Health Evaluation III
